# Transapical vs. Transaxillary Access in Transcatheter Aortic Valve Implantation: Comparative Mortality and Long-Term Outcomes Using Inverse Probability of Treatment Weighting Analysis

**DOI:** 10.3390/jcm14072235

**Published:** 2025-03-25

**Authors:** Helene Schrader, Julia M. Wiedenhofer, Sophie Berlinghof, Juliane Ducaruge, Anna Brand, Sebastian Spethmann, Ulf Landmesser, Florian Blaschke, Herko Grubitzsch, Volkmar Falk, Christoph Klein, Axel Unbehaun, Mohammad Sherif, Henryk Dreger, Tobias D. Trippel, Uwe Primessnig, Simon H. Sündermann

**Affiliations:** 1 Deutsches Herzzentrum der Charité, Department of Cardiology, Angiology and Intensive Care Medicine, Campus Virchow Klinikum–Augustenburger Platz 1, 13353 Berlin, Germanysophie.berlinghof@charite.de (S.B.); juliane.ducaruge@charite.de (J.D.); henryk.dreger@dhzc-charite.de (H.D.);; 2 Charité–Universitätsmedizin Berlin, Corporate Member of Freie Universität Berlin and Humboldt-Universität zu Berlin, Charitéplatz 1, 10117 Berlin, Germany; 3 DZHK (German Centre for Cardiovascular Research), 10785 Berlin, Germany; 4 Deutsches Herzzentrum der Charité, Department of Cardiology, Angiology and Intensive Care Medicine, Campus Charité Mitte, 13353 Berlin, Germany; 5 Deutsches Herzzentrum der Charité, Department of Cardiology, Angiology and Intensive Care Medicine, Campus Benjamin Franklin, 12203 Berlin, Germany; 6 Deutsches Herzzentrum der Charité, Department of Cardiothoracic and Vascular Surgery, 10785 Berlin, Germany

**Keywords:** transcatheter aortic valve implantation (TAVI), transapical (TAP), transaxillary TAVI (TAX), transfemoral access (TF), surgical aortic valve replacement (SAVR)

## Abstract

**Background:** Transcatheter aortic valve implantation (TAVI) is the treatment of choice for symptomatic aortic stenosis in patients with moderate to high surgical risk. When transfemoral access is unsuitable, alternative routes such as transapical (TAP) or transaxillary (TAX) routes must be considered. This study compares the in-hospital mortality and clinical outcomes of TAP vs. TAX TAVI. **Methods:** We conducted a retrospective analysis of 76 patients who underwent TAP or TAX TAVI between 2018 and 2021 at our department. Inverse probability of treatment weighting (IPTW) was used to account for baseline differences. **Results:** Among 1901 TAVI procedures, a total of 76 was selected of which TAP was performed in 34.2% (*n* = 26), and TAX in 65.8% (*n* = 50) of cases. Self-expanding CoreValve Evolut R valve prostheses were used in 96% of TAX cases, while balloon-expandable Edwards SAPIEN 3 valve prostheses were exclusively implanted in TAP cases. After IPTW adjustment, baseline characteristics, including EuroSCORE II, LVEF, and NYHA class, were comparable. TAX was associated with a higher pacemaker implantation rate (22.6% vs. 0%; *p* = 0.032), while TAP had a higher incidence of late bacteremia (13.4% vs. 1.6%; *p* = 0.027) and longer hospitalization (19 [13, 28] vs. 10 [8, 21] days; *p* = 0.016). In-hospital (0% vs. 2.1%; *p* = 0.388), 30-day (3.4% vs. 3.5%; *p* = 0.957), and 3-year mortality (6.7% vs. 4.8%; *p* = 0.709) were similar. Device implantation success was 100% in both groups. Major bleeding and vascular complications were rare. Balloon predilatation was more frequent in TAX (57.6% vs. 13.3%; *p* = 0.002). Rates of mild and moderate aortic regurgitation did not differ. **Conclusions:** In patients unsuitable for transfemoral TAVI, TAP was associated with lower pacemaker rates but longer hospitalization and increased late bacteremia compared to TAX. Both approaches showed comparable safety and efficacy, emphasizing the need for individualized access selection.

## 1. Introduction

The recommended treatment of relevant aortic valve stenosis in symptomatic elderly patients with numerous comorbidities and intermediate to high surgical risk is, in accordance with the heart team’s decision, transcatheter aortic valve implantation (TAVI) [1,2]. Conventional transfemoral access (TF) through one of the femoral arteries following multimodality imaging of the aortoiliofemoral arteries is generally considered the safest technique as it is associated with lower complication rates [3,4,5] and a reduced 1-year mortality compared to alternative access routes [2,6]. TF-TAVI has been shown to be superior to surgical aortic valve replacement (SAVR) [6,7]. However, in cases of severe peripheral artery disease, a small vessel diameter, or significant vascular tortuosity, the femoral approach may be contraindicated [8,9]. In patients in whom TF access is not feasible, alternative vascular access routes include transapical (TAP), transaxillary (TAX), direct aortic, transcarotid, transcaval, and transseptal approaches. The most frequent alternatives to TF are TAX [10] and TAP [11] access routes. Decision-making is carried out based upon the patient’s anatomy, operator preference, and local expertise in the structural heart units [11,12]. However, current evidence comparing different access strategies remains limited [13], with most available data being based on studies using first- and second-generation valves [2,10]. In the present study, we investigated in-hospital death, 30-day mortality, early clinical outcome, and complication rates of TAP versus TAX access in patients with symptomatic aortic stenosis undergoing TAVI.

## 2. Methods

**Figure 1:**
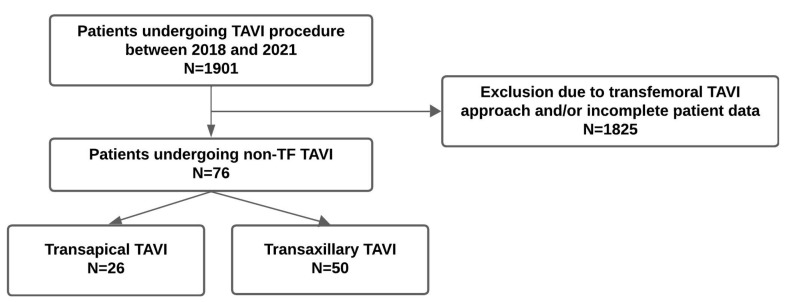
Study cohort.

**Figure 2:**
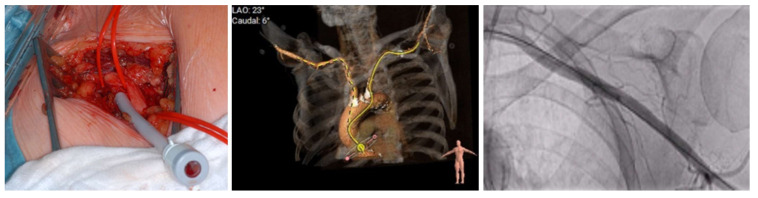
Demonstration of transaxillary TAVI access site. Operative field during transaxillary TAVI, 3D reconstruction with access via axillary artery and fluoroscopy of axillary access. Partially adapted from the chapter by Unbehaun in [14].

**Figure 3:**
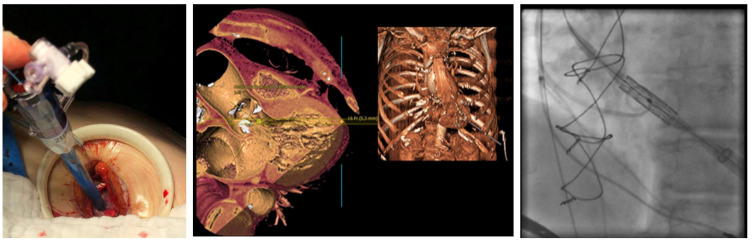
Transapical access for TAVI. Operative field during transapical TAVI, 3D reconstruction and fluoroscopy. Partially adapted from the chapter by Unbehaun in [14].

We performed a retrospective observational study and included, out of 1901 TAVI procedures in total, 76 patients with relevant symptomatic aortic stenosis who underwent TAP or TAX TAVI between 2018 and 2021 in the structural heart unit at the Department of Cardiology of the Charité–Universitätsmedizin Berlin, Campus Virchow hospital. See Figure 1. TAVI procedures were performed based on the collaborative decision of the institutional heart team following a comprehensive evaluation that included computed tomography (3mensio Medical Imaging) and echocardiography. When the TF-TAVI approach was not feasible, alternative routes (TAX or TAP) were considered according to local practice and expertise. The choice of the access route could be determined by preferred factors for TAX approach (favorable subclavian artery anatomy, aortic annulus angulation < 70° (left subclavian) or <30° (right subclavian), and no internal mammary coronary artery bypass graft) or for TAP approach (no apical LV aneurysm or thrombus, no severe aortic annular calcification, and low probability of native coronary artery occlusion). Figure 2 demonstrates the transaxillary access and Figure 3 the transapical access side for TAVI. All patients who underwent TAVI procedures via transfemoral access or had incomplete patient data at our institution between 2018 and 2021 were excluded from further analysis. While the study design did not specifically assess consecutive enrollment, all eligible cases within this timeframe were considered without additional selection bias, suggesting that they were included consecutively by default.

Valve prosthesis selection was determined by the heart team or operator, with CoreValve Evolut R used in the TAX group and Edwards Sapien 3 in the TAP TAVI approach. Device success, mortality, myocardial infarction, stroke, vascular complications, bleeding, acute kidney injury, and further clinical outcomes (like new PPM implantation and new atrioventricular block) were defined in accordance with the Valve Academic Research Consortium-2 (VARC-2) consensus document [15] and assessed during an early clinical follow-up of 30 days and a long-term evaluation 3 years after intervention. Surgical risk was estimated using the logistic EuroSCORE II (European System for Cardiac Operative Risk Evaluation). Peripheral artery disease was defined as the presence of one or more of the following: claudication, artery occlusion or >50% stenosis, amputation due to arterial disease, previous or planned intervention on the abdominal aorta, limb arteries, or carotids. Device success was defined by the correct positioning of a single prosthetic heart valve into the correct anatomical location and the intended performance of the prosthetic heart valve (no prosthesis–patient mismatch, mean aortic valve gradient <20 mm Hg or peak velocity <3 m/s, and no moderate or severe regurgitation). Early safety (at 30 days) was defined as the absence of all-cause mortality, stroke, life-threatening bleeding, acute kidney injury—stage 2 or 3 (including renal replacement therapy)—coronary artery obstruction requiring intervention, major vascular complication, or valve-related dysfunction. Clinical data were retrospectively retrieved from electronic medical records. Complete case analysis was performed for baseline characteristics, endpoints, and matching variables. All analyses were performed on anonymized datasets to ensure patient privacy and confidentiality. The study obtained ethical approval from the Charité’s ethics committee (EA4/131/23).

## 3. Statistical Analysis

Inverse probability of treatment weighting (IPTW) was employed to adjust for confounders in the analysis of access site outcomes (TAP vs. TAX) [16]. Propensity scores were estimated using a logistic regression model with sex, age, EuroScore II, and STS score as independent predictors and access site as the dependent variable. Comparative analyses were computed for both unweighted and weighted samples. Unweighted and weighted chi-squared tests and t-tests were used to compare categorical and continuous variables between the TAP and TAX groups. The normality of continuous variables was assessed using the Shapiro–Wilk test. Normally distributed variables are presented as the mean (SD), non-normally distributed variables as the median (IQR), and categorical variables as frequencies and percentages. All statistical analyses were conducted using R 4.2.3. (R Core Team 2023). The survey package was used to create the survey design object and conduct weighted analyses [17]. Descriptive statistics were generated using the tableone package [18]. The stats package was used for fitting the logistic regression model and calculating propensity scores (R Core Team, 2023).

## 4. Results

In 76 patients with symptomatic aortic stenosis, TAP or TAX TAVI was performed between 2018 and 2021. In this cohort, TAX access was used in 50 patients (65.8%), and TAP in 26 (34.2%) patients, respectively. IPTW analysis was employed on the entire cohort.

### 4.1. Study Population

In this elderly, high-risk, symptomatic population, approximately half were female. There was a high prevalence of vascular disease and other comorbidities, like arterial hypertension, chronic kidney disease, diabetes, and atrial fibrillation with a consequently high EuroSCORE II. The mean aortic valve gradient was 33.6 ± 12.9 mmHg, and the aortic peak velocity was 3.7 ± 0.6 m/s in all included patients with a normal to mild reduced LVEF (50.3% ± 14). The unweighted and weighted baseline characteristics of the study population are displayed in Table 1.

### 4.2. Procedural Characteristics

All patients underwent a successful TAP or TAX aortic valve implantation. The procedure duration was similar in both groups. Self-expanding CoreValve Evolut R valves were used in most TAX procedures, while only balloon-expandable Edwards SAPIEN 3 valves were used in TAP procedures. General anesthesia was performed in 97.3% (TAP) vs. 95.4% (TAX) (*p* = 0.651) of all cases. Balloon predilatation before valve deployment was performed more often in the TAX group (57.6% vs. 13.3%; *p* = 0.002) in keeping with differences in technique (antegrade versus retrograde prosthesis delivery) and prosthesis type. There was a significantly lower volume of contrast medium used in the TAP group, as well as a shorter duration of radiation exposure. The intraprocedural results are summarized in Table 2.

### 4.3. Procedural Outcomes, Complications, and Mortality

After the IPTW analysis, we observed no significant differences in clinical outcomes, in-hospital death (TAP 0% vs. TAX 2.1%; *p* = 0.388), 30-day mortality (TAP 3.4% vs. TAX 3.5%; *p* = 0.957), and 3-year mortality (TAP 6.7% vs. 4.8%; *p* = 0.709). Major surgical or vascular access site complications, as well as life-threatening bleeding complications, were not reported in either group.

A numerically higher, though not statistically significant, incidence of 30-day postprocedural bundle branch block was observed in the TAX group compared to the TAP group (32.4% vs. 10.9%; *p* = 0.055). This finding correlated with a significantly higher rate of new permanent pacemaker implantations in the TAX group (22.6% vs. 0%; *p* = 0.032), persisting at 3-year follow-up (26.7% vs. 2.7%; *p* = 0.0067). Hospitalization duration was significantly longer in the TAP group compared to the TAX group (19 [13, 28] vs. 10 [8, 21] days; *p* = 0.016). Additionally, a trend toward higher rates of pleural effusion was observed in the TAP group. Hemodynamic outcomes favored the TAX group, with significantly lower mean aortic valve gradients and peak velocities. However, there was a trend toward a higher incidence of moderate paravalvular leakage in the TAX group (8.2% vs. 0%; *p* = 0.078), although no case of severe regurgitation was observed post-intervention. No significant differences were noted in the incidence of prosthesis endocarditis during the early 30-day follow-up. However, bacteremia occurred more frequently in the TAP group compared to the TAX group (13.4% vs. 1.6%; *p* = 0.027). The results are summarized in Table 3 and Table 4.

## 5. Discussion

In this retrospective observational study, we compared TAX and tTAP approaches in elderly, high-risk patients with symptomatic aortic stenosis who were unsuitable for transfemoral TAVI. Our findings demonstrate that both access routes are viable alternatives when TF-TAVI is not feasible as both approaches demonstrated comparable device success rates. After an IPTW analysis to adjust for baseline differences, we observed a significantly higher rate of postprocedural pacemaker implantation in the TAX group. In contrast, the TAP approach was associated with a higher prevalence of bacteremia during long-term follow-up, though this did not impact overall mortality. No additional significant differences were found between the groups regarding mortality, major clinical outcomes, or procedural complications. While the TAX approach was associated with increased pacemaker implantation and the TAP approach with a higher incidence of long-term bacteremia, both methods demonstrated similar safety profiles and clinical outcomes. These findings underscore the importance of personalized decision-making in selecting the most appropriate TAVI access route for high-risk patients with aortic stenosis. We recognize that the lack of a direct comparison with a TF-TAVI cohort introduces a potential selection bias and limits the extrapolation of our findings to the broader TAVI population. However, our study was specifically designed to evaluate outcomes in patients for whom TF access was not feasible due to anatomical or other contraindications. Future studies including a TF control cohort would be beneficial to further contextualize our findings within the broader scope of TAVI procedures.

### 5.1. Intrahospital and Follow-Up Mortality

After conducting a weighted analysis to adjust for baseline characteristics, no significant differences were observed between the TAP and TAX TAVI groups in terms of in-hospital mortality, early mortality (beyond 30 days), or long-term mortality for up to three years of follow-up. Our findings are consistent with other recent studies where, similarly, no statistical difference was observed between TAP and TAX TAVI procedures regarding early or late mortality [19,20,21,22]. There are data from registries [23,24] and a meta-analysis [2,10] which demonstrate that the TAX approach is associated with a lower mortality rate compared to the TAP access route, while another registry from France suggested an increased late mortality in the TAX approach [12]. Our study population was at an intermediate to high risk when using the common risk score for perioperative mortality. In contrast to another recently published study [20], the EuroSCORE II was similar in both groups of our cohort, while in the study by Tomala et al., the EuroSCORE II was significantly higher in the TAP group.

### 5.2. Patient Characteristics, Clinical Outcomes, and Complications

All patients suitable for the TAP or TAX TAVI procedure were examined according to the guidelines for aortic stenosis [1]. Our cohort of elderly, symptomatic (mostly dyspnea NYHA III or IV) patients suffered from typical cardiovascular disease with an intermediate to high perioperative risk for mortality. In our study, we could not observe any differences in the TAVI device success rates between the TAP and TAX approaches, which confirmed findings from other studies [20,21,25]. In contrast to earlier published studies [2,10], the group of Tomala et al. [20], as well as our study, included only third-generation valves, namely Edwards SAPIEN 3 for the TAP approach and mostly CoreValve Evolut R for the TAX approach. Besides patient-related factors, such as comorbidities and anatomy, the operator preference determined the choice of access site in each procedure. In terms of intraprocedural characteristics, there were no differences in the operation time duration between the TAP and TAX approaches. Furthermore, most of the procedures were performed under general anesthesia. An interesting finding in our study was that the periprocedural use of contrast medium was significantly lower in TAP compared to the TAX approach and that the radiation exposure duration was also significantly lower in the TAP group. There were no differences in postprocedural complications such as acute myocardial infarction, coronary obstruction, stroke, or acute kidney injury in our study. These findings correspond to previously published studies, comparing the two approaches in non-TF TAVI [20,21]. While Ciuca et al. [21] reported a significantly higher rate of life-threatening bleeding in the TAP TAVI group compared to the TAX approach, this finding could not be confirmed in our retrospective analysis. Furthermore, the operative access site as well as vascular access-related complications were not different between both groups, which is in accordance with another recent multicenter study that reported the rates of vascular complications among TAX and TAP as 10% compared to 9.9%, respectively [21]. In a meta-analysis by Garcia et al. a decrease in vascular complications using the TAX approach compared with TAP was reported [26]. After successful device implantation in both groups, no significant differences in mild and moderate postprocedural aortic regurgitation were observed, while severe regurgitation was entirely absent in all patients. This is comparable to a previous study by Taramasso et al. [27]. They reported no significant differences between TAX and TAP with regard to postprocedural aortic regurgitation [27,28]. However, Tomala et al. demonstrated that moderate aortic regurgitation at the end of the procedure was more prevalent in the TAX group than in TAP [20]. This discrepancy compared to our study and previous studies is possibly due to the use of different valve devices. Balloon predilatation was performed slightly but significantly more often in the TAX group compared to the TAP group, a finding also reported in a previous study [20].

An important finding in our study is the significantly higher rate of permanent pacemaker implantation at both 30 days and 3 years following TAX TAVI compared to TAP TAVI (22.6% vs. 0%, *p* = 0.032, at 30 days; 26.7% vs. 2.7%, *p* = 0.0067, over three years of follow-up). While absolute numbers indicated a higher incidence of postprocedural bundle branch block in the TAX group (29.2% vs. 13%), this difference did not reach statistical significance (*p* = 0.32). This result is line with the evidence from previous studies, which have shown that early pacemaker implantation was more frequent after TAX than TAP procedures [10]. It was also reported that pacemaker implantation rates tend to be higher with CoreValve Evolut R valves compared with Edwards SAPIEN 3 [29]. Our study also identified a higher prevalence of late-term bacteremia in the TAP group (13.4% vs. 1.6%; *p* = 0.027). This observation aligns with the findings obtained by Bjursten et al., who identified the TAP approach as an independent risk factor for late-term endocarditis, diagnosed more than one year post-intervention [30].

Finally, we could demonstrate in our study that the total time for hospitalization was significantly longer in the TAP group compared to the TAX group (19 [13, 28] vs. 10 [8, 21]; *p* = 0.016) which could be associated with the higher rate of pulmonary congestion and pleural effusion after TAP access. Similar findings were reported by Price et al. They demonstrated that TAX-TAVI leads to a shorter ventilator duration, which consecutively results in a reduction in hospital length of stay compared to TAP-TAVI [31].

## 6. Limitations

There are some important limitations regarding our work. First, this study was designed as a single-center, retrospective observational study as it relies on the analysis of existing medical records, introducing potential biases and limitations related to the collection and availability of data. Second, the sample size of our study was relatively small. However, due to the investigation and the comparison of TAP versus TAX TAVI approaches, the sample size was to be expected. Due to the limited sample size, our study is underpowered to conduct a robust subgroup analysis stratified by aortic stenosis physiology (LF/LG vs. HG) and prosthesis type. Future studies with larger cohorts would be needed to explore these variables in greater detail and assess their potential impact on clinical outcomes. Patients in our study were not randomly assigned to the treatment groups, and although we attempted to address this limitation through an IPTW analysis, there is still the possibility of hidden confounders that may have introduced bias into our results. Furthermore, our dataset does not include systematic information on concomitant medical therapies, which may have influenced patient outcomes. Given the growing body of evidence on the cardiovascular benefits of SGLT2 inhibitors and other medical therapies in high-risk populations, future studies incorporating detailed pharmacological data will be essential to assess their impact in TAVI patients.

## 7. Conclusions

In this study of patients unsuitable for TF-TAVI, we found that the TAP approach was associated with a lower rate of postinterventional pacemaker implantations, but longer hospital stays in comparison to the TAP access route. Additionally, our analysis of both early and long-term outcomes revealed a higher prevalence of late-term bacteremia in the TAP group. Despite these differences, both approaches demonstrated comparable efficacy and safety profiles, with no significant differences in in-hospital, early 30-day, and late 3-year mortality; clinical outcomes; or other major complications. Given the study’s design and inherent limitations, these findings should be interpreted with caution. In our investigation both access routes demonstrated to be viable alternatives for patients ineligible for TF- TAVI. However, the choice should be based on an individualized assessment of patient-specific risk factors and anatomical considerations. Further studies with larger cohorts and prospective designs are needed to refine patient selection criteria and optimize outcomes for patients requiring alternative TAVI access routes.

## Figures and Tables

**Table 1:** Baseline parameters before and after IPTW.

	Before IPTW			After IPTW		
	TAP (N = 26)	TAX (N = 50)	*p*-Value	TAP (N = 26)	TAX (N = 50)	Weighted *p*-Value
Female	11 (42.3%)	25 (50.0%)	0.693	11 (41.3%)	20 (40.4%)	0.950
Age (years)	75.9 (6.69)	81.8 (6.00)	<0.001 *	78.1 (1.03)	78.0 (2.28)	0.989
Arterial hypertension	23 (88.5%)	47 (94.0%)	0.688	23 (90.3%)	48 (95.6%)	0.350
BMI (kg/m^2^)	26.3 (4.35)	24.1 (3.33)	0.031 *	26.4 (0.93)	24.78 (0.92)	0.226
NYHA I, II	4 (20.0%)	6 (15.8%)	0.97	3 (10.6%)	3 (5.6%)	0.445
NYHA III, IV	16 (80.0%)	32 (84.2%)	0.97	20 (77.9%)	43 (86.4%)	0.445
Diabetes mellitus	7 (26.9%)	12 (24.0%)	1	2 (9.3%)	5 (10.7%)	0.471
Coronary heart disease	22 (84.6%)	43 (86.0%)	1	22 (83.9%)	44 (87.7%)	0.660
History of atrial fibrillation	12 (46.2%)	21 (42.0%)	0.918	12 (45.8%)	17 (33.0%)	0.326
Renal insufficiency	15 (57.7%)	20 (40.0%)	0.22	16 (62.8%)	21 (42.7%)	0.157
Euro Score II	10.5 [0.810, 32.0]	7.20 [1.94, 37.2]	0.259	10.55 [1.95, 18.00]	7.2 [1.03, 18.11]	0.951
STS Score	4.97 [1.60, 16.9]	6.00 [1.89, 18.9]	0.25	5.14 [3.98, 7.4]	6.00 [4.4, 8.0]	0.771
LVEF (%)	47.1 (13.7)	52.1 (14.0)	0.183	47.5 (2.77)	46.4 (5.22)	0.857
Aortic valve area (cm^2^)	0.724 (0.197)	0.710 (0.161)	0.775	0.723 (0.046)	0.682 (0.059)	0.577
Aortic valve mean gradient (mmHG)	34.3 (14.3)	33.2 (12.4)	0.808	35.16 (3.21)	31.48 (2.24)	0.353
Aortic valve peak gradient (mmHG)	57.1 (22.0)	53.8 (17.6)	0.6	58.49 (5.33)	53.72 (3.15)	0.445
AV V_max_ (m/s)	3.73 (0.688)	3.63 (0.571)	0.597	3.77 (0.170)	3.55 (0.105)	0.288

Values are displayed as mean (standard deviation), absolute numbers (percent frequencies), and median [IQR]. IPTW, inverse probability of treatment weighting; BMI, body mass index; NYHA, New York Heart Association; LVEF, left ventricular ejection fraction; AV V_max_, atrioventricular peak velocity; TAP, transapical access; TAX, transaxillary access. * Significant, *p* < 0.5.

**Table 2:** Procedural details before and after IPTW.

	Before IPTW			After IPTW		
	TAP (N = 26)	TAX (N = 50)	*p*-Value	TAP (N = 26)	TAX (N = 50)	Weighted *p*-Value
Procedural duration (min)	138 (50.1)	139 (40.7)	0.648	137 (10.67)	138 (6.34)	0.998
Peri-operative anaethesia						
Analog sedation	1 (4.0%)	3 (7.0%)	1	1 (2.7%)	2 (4.6%)	0.651
General anesthesia	24 (96.0%)	40 (93.0%)	1	25 (97.3%)	48 (95.4%)	0.651
Valve prosthesis system						
CoreValve Evolut	0 (0%)	47 (94.0%)	NA	0 (0%)	47 (94.0%)	NA
Edwards Sapien 3	26 (100%)	2 (4.0%)	NA	26 (100%)	2 (4.0%)	NA
Portico	0 (0%)	1 (2.0%)	NA	0 (0%)	1 (2.0%)	NA
Contrast medium volume (mL)	90.4 (61.7)	129 (41.6)	<0.001 *	97.8 (16.07)	134.57 (7.45)	0.041 *
Radiation exposure (min)	7.77 (4.50)	16.6 (8.52)	<0.001 *	8.24 (1.12)	16.06 (1.14)	<0.001 *
Radiation dose (Gy/cm^2^)	23.9 (15.8)	30.0 (18.6)	0.225	25.11 (3.48)	34.61 (3.88)	0.073
Valze size (mm)	25.7 (2.13)	28.2 (2.48)	<0.001 *	25.57 (0.42)	28.68 (0.65)	<0.001 *
Valvuloplastie	3 (11.5%)	23 (47.9%)	0.004 *	3 (13.3%)	29 (57.6%)	0.002 *
Valve implantation success	26 (100%)	50 (100%)	NA	26 (100%)	50 (100%)	NA

Values are displayed as mean (standard deviation), absolute numbers (percent frequencies), and median [IQR]. NA, not applicable; IPTW, inverse probability of treatment weighting; TAP, transapical access; TAX, transaxillary access. * Significant, *p* < 0.5.

**Table 3:** Early clinical outcomes.

	Before IPTW			After IPTW		
	TAP (N = 26)	TAX (N = 50)	*p*-Value	TAP (N = 26)	TAX (N = 50)	Weighted *p*-Value
Safety composite	2 (7.7%)	4 (8.0%)	1	2 (9.5%)	3 (6.2%)	0.649
In-hospital mortality	0 (0%)	1 (2.0%)	1	0 (0%)	1 (2.1%)	0.388
30-day mortality	1 (3.8%)	2 (4.0%)	1	1 (3.4%)	2 (3.5%)	0.957
Postprocedural myocardial infarct						
<72 h periprocedural	0 (0%)	0 (0%)	NA	0 (0%)	0 (0%)	NA
>72 h postprocedural	0 (0%)	1 (2.0%)	1	0 (0%)	1 (1.4%)	0.393
AKIN						
1	1 (3.8%)	1 (2.0%)	1	1 (1.7%)	1 (1.4%)	0.921
2	0 (0%)	0 (0%)	NA	0 (0%)	0 (0%)	NA
3	2 (7.7%)	1 (2.0%)	0.532	2 (9.5%)	1 (1.4%)	0.087
Stroke (non-diabling or disabling)	0 (0%)	2 (4.0%)	0.8	0 (0%)	2 (3.3%)	0.244
Bleeding type						
Life-threatening	0 (0%)	1 (2.0%)	1	0 (0%)	1 (2.1%)	0.388
Major	2 (7.7%)	0 (0%)	0.205	1 (5.4%)	0 (0%)	0.140
Minor	1 (3.8%)	4 (8.0%)	0.87	2 (7.2%)	8 (15.1%)	0.492
Bleeding (all types)	3 (11.5%)	5 (10.0%)	1			0.684
Postprocedural higher AV-block	0 (0%)	5 (10.0%)	0.252	0 (0%)	9 (17.7%)	0.091
Postprocedural BB block	3 (12.0%)	15 (30.0%)	0.152	3 (10.9%)	16 (32.4%)	0.055
New pacemaker device	0 (0%)	8 (16.0%)	0.085	0 (0%)	11 (22.6%)	0.032 *
Coronary obstruction	0 (0%)	1 (2.0%)	1	0 (0%)	1 (1.4%)	0.393
Major vascular complication	0 (0%)	0 (0%)	NA	0 (0%)	0 (0%)	NA
Peri-interventional BB block						
Left BB block	5 (20.0%)	7 (14.6%)	0.737	4 (14.4%)	7 (13.5%)	0.946
Right BB block	1 (4.0%)	1 (2.1%)	1	2 (7.5%)	1 (1.7%)	0.247
Paravalvular leakage						
1	7 (26.9%)	20 (40.0%)	0.444	7 (26.6%)	22 (44.2%)	0.189
2	0 (0%)	5 (10.0%)	0.252	0 (0%)	4 (8.2%)	0.078
3	0 (0%)	0 (0%)	NA	0 (0%)	0 (0%)	NA
Access site complication type						
Major	0 (0%)	0 (0%)	NA	0 (0%)	0 (0%)	NA
Minor	1 (3.8%)	1 (2.0%)	1	1 (5.0%)	1 (1.3%)	0.298
Pleural effusion	4 (15.4%)	3 (6.0%)	0.652	4 (15.7%)	3(4.9%)	0.070
Prosthesis endocarditis	1 (3.8%)	1 (2.0%)	1	1 (3.4%)	1 (1.7%)	0.635
Total hospital days	19.0 [5.00, 54.0]	9.50 [5.00, 32.0]	0.002 *	19.0 [13.00, 28.00]	10 [8.00, 21.00]	0.016 *

Values are displayed as mean (standard deviation), absolute numbers (percent frequencies), and median [IQR]. NA, not applicable; IPTW, inverse probability of treatment weighting; AV, atrioventricular; BB, bundle branch; TAP, transapical access; TAX, transaxillary access. * Significant, *p* < 0.5.

**Table 4:** Long-term outcomes after 3 years.

	Before IPTW			After IPTW		
	TAP (N = 26)	TAX (N = 50)	*p*-Value	TAP (N = 26)	TAX (N = 50)	Weighted *p*-Value
Safety composite	3 (11.5%)	4 (8.0%)	0.930	4 (13.8%)	5 (9.0%)	0.518
All-cause death	2 (7.7%)	3 (6.0%)	1	2 (6.7%)	2 (4.8%)	0.709
Rehospitalization	14 (53.8%)	35 (70%)	0.253	10 (37.0%)	22 (43.0%)	0.667
Cardiac decompensation	0 (0.0%)	3 (6.0%)	0.513	0 (0.0%)	8 (15.2%)	0.152
Resusication	1 (3.8%)	1 (2.0%)	1	1 (3.6%)	1 (2.1%)	0.702
Postprocedural myocardial infarct	0 (0%)	1 (2.0%)	1	0 (0.0%)	1 (1.4%)	0.393
AKIN						
1	5 (19.2%)	7 (14.0%)	0.794	4 (16.1%)	14 (28.8%)	0.335
2	1 (3.8%)	1 (2.0%)	1	4 (1.6%)	1 (1.5%)	0.975
3	2 (7.7%)	1 (2.0%)	0.556	2 (9.0%)	1 (1.1%)	0.087
Stroke (non-diabling or disabling)	1 (3.8%)	2 (4.0%)	1	1 (3.2%)	2 (3.2%)	0.988
Bleeding type						
Life-threatening	0 (0.0%)	1 (2.0%)	1	0 (0.0%)	1 (2.0%)	0.388
Major	2 (7.7%)	0 (0.0%)	0.210	1 (5.3%)	0 (0.0%)	0.140
Minor	1 (3.8%)	4 (8.0%)	0.837	2 (7.1%)	8 (15.4%)	0.492
Higher-grade AV block	1 (3.8%)	5 (10.0%)	0.620	1 (5.0%)	9 (17.9%)	0.223
Onset of atrial fibrillation	3 (11.5%)	1 (2.0%)	0.220	2 (8.9%)	1 (2.0%)	0.164
Pacemaker device	1 (3.8%)	10 (20.0%)	0.120	1 (2.7%)	13 (26.7%)	0.0067 *
Valve intervention	1 (3.8%)	0 (0.0%)	0.738	1 (3.1%)	0 (0.0%)	0.265
Coronary angiography	1 (3.8%)	2 (4.0%)	1	1 (3.1%)	2 (4.1%)	0.815
Coronary obstruction	1 (3.8%)	1 (2.0%)	1	1 (3.1%)	1 (1.4%)	0.578
Bacteremia	4 (15.4%)	1 (2.0%)	0.081	4 (13.4%)	1 (1.6%)	0.027 *

Values are displayed as mean (standard deviation), absolute numbers (percent frequencies), and median [IQR]. IPTW, inverse probability of treatment weighting; AV, atrioventricular; BB, bundle branch; TAP, transapical access; TAX, transaxillary access. * Significant, *p* < 0.5.

## Data Availability

The authors will provide the raw data of this article without further reservation.
